# P-1148. Invasive Infections by Carbapenemase-producing Enterobacterales and *Pseudomonas aeruginosa* in Children

**DOI:** 10.1093/ofid/ofae631.1334

**Published:** 2025-01-29

**Authors:** Claudia Inés I Cazes, Estefania Biondi, Romina Lanfranchi, Aldo Cancellara, Adriana Procopio, Carolina Ordoñez, Gustavo Cesar Ezcurra, Carolina Aró, Ximena S Juarez, Ezequiel Flores, Valeria Cames, Patricia Dondoglio, Rosana Pereda, Hugo Granchetti, Antonella Pezzaniti, Daniela Satragno, Miryam Vazquez, Eduardo L López

**Affiliations:** Hospital General de Niños Dr. Ricardo Gutiérrez, Buenos Aires, Buenos Aires, Argentina; Hospital de Niños R. Gutiérrez, Buenos Aires, Buenos Aires, Argentina; Hospital de Niños Ricardo Gutierrez, CABA, Ciudad Autonoma de Buenos Aires, Argentina; Hospital de Niños P. de Elizalde, Buenos Aires, Buenos Aires, Argentina; Hospital de Niños R. Gutiérrez, Buenos Aires, Buenos Aires, Argentina; Hospital de Niños R. Gutiérrez, Buenos Aires, Buenos Aires, Argentina; Hospital Orlando Alassia, Santa Fe, Santa Fe de la Vera Cruz, Santa Fe, Argentina; Hospital Orlando Alassia. Santa Fe, Santa Fe de la Vera Cruz, Santa Fe, Argentina; Hospital de Niños P. de Elizalde, Buenos Aires, Buenos Aires, Argentina; Hospital de Niños R. Gutiérrez, Buenos Aires, Buenos Aires, Argentina; Hospital de Niños R. Gutiérrez, Buenos Aires, Buenos Aires, Argentina; Hospital de Niños P. de Elizalde, Buenos Aires, Buenos Aires, Argentina; Hospital de Niños P. de Elizalde, Buenos Aires, Buenos Aires, Argentina; Pharmacy and Biochemistry, University of Buenos Aires, Buenos Aires, Buenos Aires, Argentina; Pharmacy and Biochemistry, University of Buenos Aires, Buenos Aires, Buenos Aires, Argentina; Hospital de Niños R. Gutiérrez, Buenos Aires, Buenos Aires, Argentina; Hospital de Niños R. Gutiérrez, Buenos Aires, Buenos Aires, Argentina; Pediatric Infectious Disease Program, Hospital de Niños Ricardo Gutiérrez, Universidad de Buenos Aires, Ciudad Autónoma de Buenos Aires, Buenos Aires, Argentina

## Abstract

**Background:**

Enterobacterales and *Pseudomonas aeruginosa* cause invasive infections and their spread of antimicrobial resistance is of great concern in the worldwide scientific community. Carbapenemases represent the main mechanism of resistance in Enterobacterales and can spread horizontally through mobile genetic elements. Clinical evidence suggests that mortality would be related to inadequate initial empirical treatment.

**Figure:**
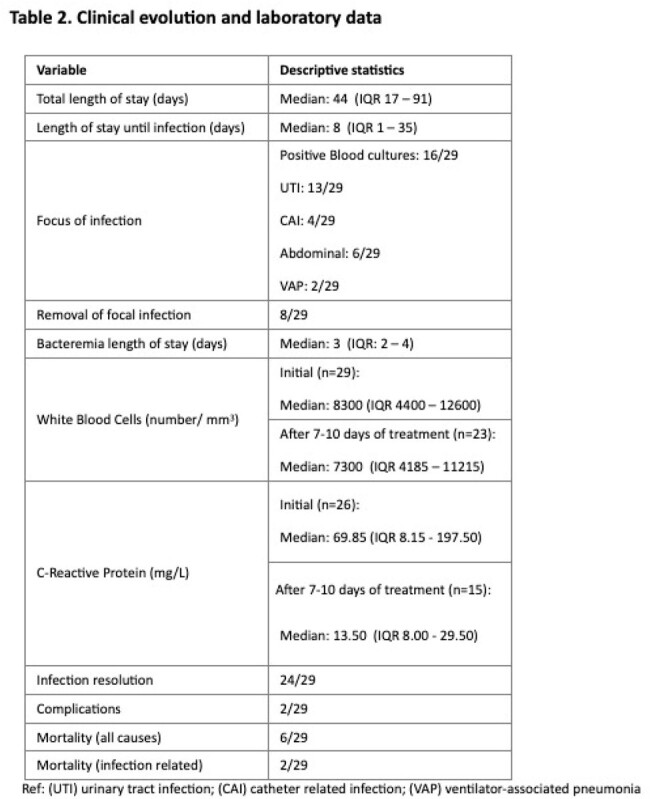

**Methods:**

A prospective observational study was carried out in three tertiary care pediatric hospitals in Argentina, since 12/1/2021 to 11/30/2022, including children between 0 to 18 years of age with a positive culture for carbapenemase-producing Enterobacterales or *P. aeruginosa* isolated from a sterile site and/or tracheal aspirate with pulmonary radiological confirmation.

**Results:**

We enrolled 29 patients, 62% male. Median age: 25 months (IQR 5-127). Median length of hospitalization: 44 days (IQR 17-91). Comorbidities: 93%, oncohematological disease: 40%; 62% (18/29) were admitted to Intensive Care Unit. The most frequent isolation was *Klebsiella pneumoniae:* 62% (18/29), *Escherichia coli* 17% (5/29), *Enterobacter cloacae* 10% (3/29), *Serratia marcescens* 7% (2/29), *P. aeruginosa* 7% (2/29). Bacteremia: 55% (16/29). Most frequent site of infection: urinary tract 44.8%. Metallobetalactamase (MBL) was detected in 48% (14/29), KPC: 34% (10/29), OXA: 14% (4/29). Initial empirical treatment: meropenem 53% (16/29). Adjustment of initial empirical treatment at 48 hours: 79% (23/29) according to cultures and antibiogram. Ceftazidime/avibactam: 48.2%; aztreonam combined in MBL strains. Extended infusion meropenem: 24% (7/29) if MIC ≤ 8 ug/ml. Overall mortality: 20.6%, in 6.8% it was related to infection.

**Conclusion:**

In recent years we have seen an increase in antimicrobial resistance. Metallobetalactamases were the most frequently detected in patients hospitalized in critical areas. Resistance data in Argentina showed *K. pneumoniae* as one of the most prevalent bacteria in multidrug-resistant infections. Carbapenemes in high doses and extended infusions plus other antibiotics were useful to treat strains with low MIC values. Ceftazidime/avibactam was a good option for KPC and some OXA infections. Aztreonam in combined use was indicated for MBL strains.

**Disclosures:**

**All Authors**: No reported disclosures

